# Serum autoantibody profiling of oral squamous cell carcinoma patients reveals NUBP2 as a potential diagnostic marker

**DOI:** 10.3389/fonc.2023.1167691

**Published:** 2023-09-22

**Authors:** Riaz Abdulla, Jofy Devasia Puthenpurackal, Sneha M. Pinto, Punchappady Devasya Rekha, Yashwanth Subbannayya

**Affiliations:** ^1^ Department of Oral Pathology and Microbiology, Yenepoya Dental College, Yenepoya (Deemed to be University), Mangalore, India; ^2^ Yenepoya Research Centre, Yenepoya (Deemed to be University), Mangalore, India; ^3^ School of Biosciences, Faculty of Health and Medical Sciences, University of Surrey, Guildford, United Kingdom

**Keywords:** biomarkers, early detection, liquid biopsy, autoantibodies, screening biomarkers

## Abstract

**Introduction:**

Oral Squamous Cell Carcinoma (OSCC), a common malignancy of the head and neck region, is frequently diagnosed at advanced stages, necessitating the development of efficient diagnostic methods. Profiling autoantibodies generated against tumor-associated antigens have lately demonstrated a promising role in diagnosis, predicting disease course, and response to therapeutics and relapse.

**Methods:**

In the current study, we, for the first time, aimed to identify and evaluate the diagnostic value of autoantibodies in serum samples of patients with OSCC using autoantibody profiling by an immunome protein array. The utility of anti-NUBP2 antibody and tissue positivity in OSCC was further evaluated.

**Results and discussion:**

We identified a total of 53 autoantibodies with significant differential levels between OSCC and control groups, including 25 that were increased in OSCC and 28 that were decreased. These included autoantibodies against Thymidine kinase 1 (TK1), nucleotide-binding protein 2 (NUBP2), and protein pyrroline-5-carboxylate reductase 1 (PYCR1), among others. Immunohistochemical validation indicated positive staining of NUBP2 in a large majority of cases (72%). Further, analysis of OSCC data available in TCGA revealed higher NUBP2 expression correlated with better disease-free patient survival. In conclusion, the differential serum autoantibodies identified in the current study, including those for NUBP2, could be used as potential biomarkers for early diagnosis or as screening biomarkers for OSCC pending investigation in a larger cohort.

## 1 Introduction

Cancers of the lip and the oral cavity constitute the 16^th^ most common neoplasms in the world, with 377,713 new cases (Age-standardized incidence: 4.1) and 177,757 mortalities (Age-standardized incidence: 1.9) estimated in 2020 (1). The highest incidences of these cancers have been reported in Papua New Guinea, Pakistan, Latvia, India, and Bangladesh (2). Oral squamous cell carcinoma (OSCC) cases constitute 90% of lip and oral cavity cancers. OSCC has a relatively low survival rate, with increasing incidences reported in South-Central Asia and parts of Oceania (2). Alcohol and tobacco consumption constitute important risk factors for the development of OSCC (3, 4). Tobacco consumption, both in the form of cigarette smoke (5, 6) as well as smokeless tobacco, has been reported as a significant risk factor (4, 7). Further, infection with the human papillomavirus has also been implicated as a risk factor in a subset of OSCC cases (8). Several oral mucosal diseases, including leukoplakia, erythroplakia, oral submucous fibrosis (OSMF), and oral lichen planus, amongst others, have been grouped as oral potentially malignant disorders (OPMDs), and these have an increased risk of malignant transformation to OSCC (9). Cessation of smoking/alcohol consumption and early diagnosis/screening have been suggested to significantly reduce mortality from OSCC (10, 11). However, despite gold-standard imaging techniques, including MRI and CT, as well as standard incisional biopsy being used for diagnosis (12), approximately 60% of OSCC patients present with advanced stages of disease (III/IV) at the time of diagnosis (13). This necessitates the development of efficient and accurate molecular diagnostics methods that could be used for early diagnosis.

Body fluids constitute attractive targets for liquid biopsies to identify biomolecules capable of indicating a tumor’s state. Several high-throughput proteomics technologies, including mass spectrometry, and antibody/antigen arrays, are increasingly being used to characterize and quantitate liquid biopsy samples (14). Multiple groups have used body fluids such as serum (15, 16), saliva (17, 18) and FFPE tissue (19) to identify potential biomarkers for OSCC. Over recent years, tumor autoantibodies (TAAbs) have gained attention as potential cancer biomarkers that can be extracted from serum using minimally invasive sampling. TAAbs have been observed in several types of cancers, including lung (20, 21), gastric (22), hepatocellular (23), breast (24), ovarian (25), and prostate (26) cancers, among others. Further, increased autoantibody levels have been observed in the early stages of cancer (21, 22). In addition, AAbs are stable (27) and persistent even after the antigen is no longer detected (25). These findings suggest the utility of AAbs as potential markers for early diagnosis, screening, and prognosis.

In the current study, we carried out autoantibody profiling of oral cancer using an immunome protein array that allows for the detection of autoantibodies in patient serum samples, making it an effective tool for biomarker discovery. Using a cohort of 20 patient samples and 20 control cases, significantly altered levels of autoantibodies against 53 proteins were identified. Of these, 25 demonstrated increased levels and 28 decreased levels in oral cancer patients. Further, we evaluated the correlation between anti-NUBP2 antibody and tissue positivity in OSCC.

## 2 Materials and methods

### 2.1 Patient specimens

A total of 40 serum samples, including 20 samples from patients diagnosed with oral cancer and 20 samples from healthy volunteers, were collected and stored at −80°C after obtaining study approval from the Yenepoya (Deemed to be University) Ethics Committee Mangalore (#2013/149 dated 24/07/2013). Informed consent was obtained from patients for the samples collected. The details on these samples are provided in **Supplementary Table 1**. Archival Formalin-fixed Paraffin-embedded (FFPE) sections were obtained for another 25 cases of OSCC to carry out immunohistochemical validation. The FFPE blocks were collected after obtaining study approval from the Yenepoya (Deemed to be University) Ethics Committee Mangalore (#2016/239 dated 12/11/2016). The details for these samples are provided in **Supplementary Table 2**.

### 2.2 Immunome protein microarray-based autoantibody profiling

The Sengenics Immunome Protein Array platform was used for the high-throughput quantification of autoantibodies (Sengenics Corporation LLC, https://sengenics.com/i-ome-array/). The immunome array is a patented technology that uses a biotin carboxyl carrier protein (BCCP) domain affinity tag. The experiment was performed by the Sengenics Corporation LLC. Serum samples were thawed for 30 minutes using a shaking incubator at 20°C. A total of 22.5 μL of each sample was diluted with 4.5 mL of Serum Assay Buffer (SAB, 0.1% v/v Triton, 0.1% w/v BSA in PBS) and mixed. The sera were aspirated from below the formed lipid layer at the top. The immunome array was removed from the storage buffer, washed with 200 mL cold SAB, and shaken on an orbital shaker at 50 rpm for 5 minutes. The slide was then placed in a slide hybridization chamber with individual sera and incubated on a horizontal shaker at 50 rpm for 2 hours at 20°C. Post-incubation, the arrays were rinsed with SAB for 20 minutes on the shaker at 50 rpm at room temperature. The arrays were then labeled with a hybridization solution containing a mixture of Cy3- rabbit antihuman IgG (Dako Cytomation) solution diluted 1:1000 in SAB for 2 hours at 50 rpm at 20°C. Post-labeling, the arrays were washed in SAB followed by water and dried for 2 min at 240g at room temperature. Slides were then stored at room temperature until scanning. The hybridization signals were measured with a microarray laser scanner (Agilent Technologies) at 10μm resolution. Fluorescence levels were detected, and data were acquired from the microarray scanner in a raw .tiff format and subjected to further analysis.

### 2.3 Data analysis

Data analysis was carried out using the Agilent Feature Extraction software with customized scripts. The data for samples were matched in pairs between case and control groups. Automatic spot identification and detection were carried out using GenePix Pro 7 software. Data mining and analysis for quality control and biomarkers identification were done using customized scripts created in R and Perl. Quality control on raw and normalized data was carried out to verify the quality of the protein array data before proceeding with the data analysis using four methods. These included (i) Calculating the median of the raw signal intensities from the quadruplet protein spots on each slide (i.e. each sample), (ii) Subtracting median background signals from the median raw median signal intensities, (iii) Inspecting signal intensities of two positive controls: IgG and Cy3BSA, (iv) Quantile normalization of data with the exclusion of control proteins, i.e. normalization of 1631 protein spots across all samples, and (v) Calculating the percentage of coefficient of variant (CV%) of intra-protein, intra-slide, and inter-array to determine the variations between the quadrupled signal intensity for each protein spot on the slide.

The identification and ranking of protein biomarkers were made using a penetrance-based fold change. A penetrance-based fold change measures the likelihood that a given raw fold change is true, thus increasing the significance and reliability of the results. Subsequently, quantile normalization of data with the exclusion of control proteins, i.e., normalization of only 1631 protein spots across all samples, was carried out. Further, individual fold changes for both case (H_case_) and control (H_control_) samples were calculated by dividing each normalized data by the mean of each protein across all samples. Penetrance frequencies were calculated for each protein for both case (Frequency_case_) and control (Frequency_control_). Penetrance Fold Changes for both case (PFC_case_) and control (PFC_control_) were calculated for each protein by dividing H_case_ by H_control_ and H_control_ by H_case,_ respectively. P-value was calculated using a Student T-test for the two, and overall fold-change was calculated by dividing the mean of each protein across all case samples, μ(H_case_), with the mean of each protein across all control samples μ(H_control_). Significantly changing markers were identified and ranked based on (i) P-value < 0.05, (ii) Penetrance Fold Change Difference of ≥ 2 and Frequency Differential ≥ 1 for upregulated markers, (iii) Penetrance Fold Change Difference of ≤ -2 and Frequency Differential ≤ -1. for downregulated markers, (iv) Frequency Percentage of 10% in both cases and controls.

### 2.4 Immunohistochemical validation

Archival paraffin-embedded tissue blocks of confirmed OSCC cases from the Department of Oral Pathology and Microbiology, Yenepoya Dental College, Mangalore, were used for the immunohistochemical validation. The blocks were used to prepare tissue microarray (TMA) mother blocks (Lab Surgpath, Mumbai, India). The TMA mother blocks were subjected to serial sectioning to prepare sections of 3-5µ thickness and fixed on glass slides. Each section was used for Haematoxylin and Eosin (H-&-E) staining and immunostaining. Normal parietal cells located in the gastric gland found in the lining of the fundus from archival FFPE blocks were taken as a control. We used the parietal cell controls as we were unable to obtain normal cells from patients due to ethical reasons. The antibody datasheet from the manufacturer (https://datasheets.scbt.com/sc-376784.pdf) showed positive staining with glandular cells and not parietal cells; therefore we chose to use these cells as a negative control.

Immunohistochemistry was performed on OSCC tissue microarrays as well as controls for NUBP2. The mouse monoclonal anti-NUBP2 antibody was purchased from Santa Cruz Biotechnology Inc. (sc-376784) and used at 1:50 dilution. Briefly, tissue microarrays were deparaffinized in xylene. Heat-induced antigen retrieval was carried out by placing the slides in Tris EDTA epitope retrieval buffer in a pressure cooker until full pressure was released three times. The slides were removed once the pressure was released and rinsed in distilled water, followed by washing using a wash buffer (Immuno Wash Buffer (25X), Tris Buffered Saline with Tween 20, Pathnsitu, CA, USA) and drained. Endogenous peroxidase activity was quenched by treating the sections with hydrogen peroxide (Poly Excel, PathnSitu, CA, USA) for 20 minutes. The slides were then rinsed in distilled water for 2 minutes and washed twice for 3 minutes, each with wash buffer.

The sections were incubated overnight at 4°C in a humidified chamber with the primary antibody. After washing twice for 5 minutes in wash buffer, the slides were incubated with horse radish peroxidase-conjugated anti-mouse secondary antibody (# sc-516102 - m-IgGκ BP-HRP, Santa Cruz Biotechnology Inc.). The slides were treated with DAB chromogen (Poly Excel Stunn DAB, PathnSitu, CA, USA) solution for 5 minutes at room temperature and counterstained with Mayer’s hematoxylin for 2 minutes. Two investigators independently analyzed all IHC slides along with corresponding H&E sections. The immunohistochemically stained sections were scanned under 10X using a bright-field binocular microscope. Cytoplasmic/membranous staining was considered as a positive immune reaction for NUBP2. The IHC-stained slides were graded positive if >5% of cells were stained and negative if less than <5% of the cells were stained. The expression of NUBP2 was correlated with clinicopathological parameters like age group, gender, tumor location, tumor size, and nodal metastasis. Fischer’s exact test was performed to find the association between various parameters like age group, gender, tumor location, tumor size, and nodal metastasis with NUBP2 expression.

### 2.5 Analysis of NUBP2 expression in TCGA data and survival analysis

Expression of proteins with observed upregulated autoantibodies was queried in the GDC TCGA Head and Neck Cancer transcriptome data using the Xena Functional Genomics Explorer (https://xenabrowser.net/, accessed June 17, 2022) (28). We chose to compare with the TCGA HNSCC cohort of 528 samples as the majority of the cases are resected from the oral cavity (72.4%) (29).

The prognostic value of NUBP2 and other upregulated candidates in Head-Neck Squamous Cell transcriptome datasets was investigated by survival analysis using the GEPIA server (http://gepia.cancer-pku.cn) (30).

### 2.6 Species conservation analysis

The sequence conservation analysis for NUBP2 was carried out as previously described (31). Orthology data for all human genes were obtained from Homologene (Release 68, downloaded on October 4, 2018, from https://www.ncbi.nlm.nih.gov/homologene). RefSeq accessions of NUBP2 orthologs were retrieved from NCBI gene (https://www.ncbi.nlm.nih.gov/gene) and protein sequences were fetched using Batch Entrez (https://www.ncbi.nlm.nih.gov/sites/batchentrez). Alignment of protein sequences was carried out using Clustal Omega (https://www.ebi.ac.uk/Tools/msa/clustalo/) using default settings and the dendrogram (.dnd) obtained was visualized with Interactive Tree of Life (https://itol.embl.de/) with custom colors and tracks. Further, ortholog counts for all genes in the Homologene database were obtained, and the Taxonomy ID for each gene was mapped to the species type. The densities of ortholog counts of NUBP2 were plotted against the density of ortholog counts for all human genes in the background using R (v4.0.2) (https://cran.r-project.org/).

## 3 Results

**Figure 1:**
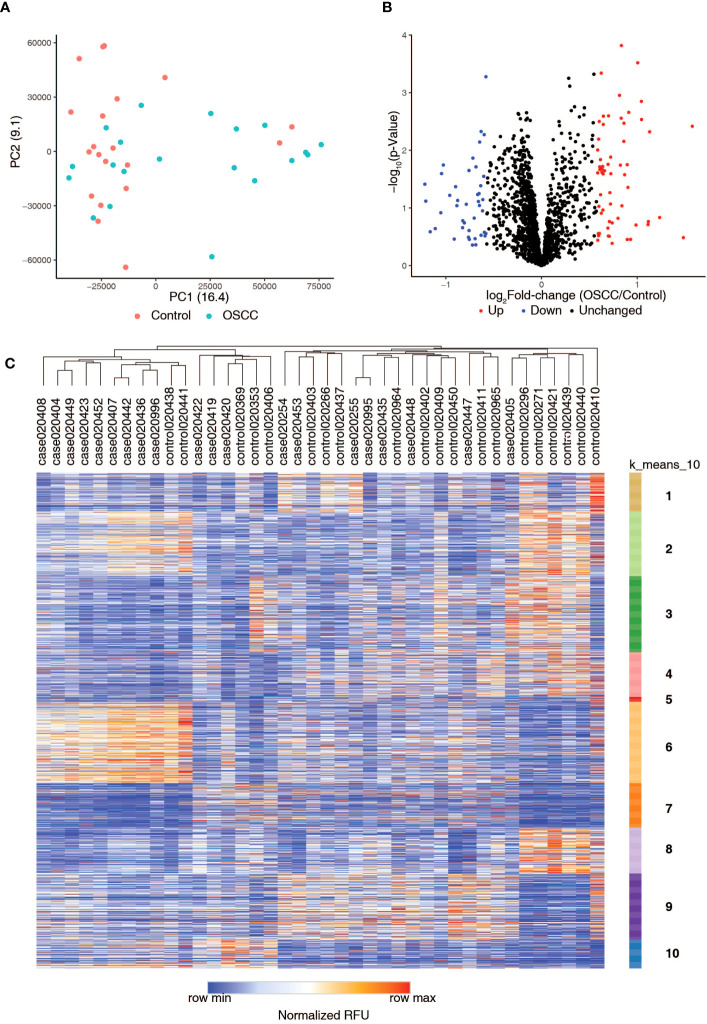
Summary of autoantibody profiling of serum samples from healthy volunteers (Control) and serum samples from patients with oral squamous cell carcinoma (OSCC). **(A)** Principal Component Analysis (PCA) plot shows the distribution of Control and OSCC samples. **(B)** Volcano plot showing differential levels of autoantibodies in oral squamous cell carcinoma samples as compared to controls. **(C)** Heatmap showing the autoantibody profiling data along with k-means clustering of the data.

Autoantibody profiling of serum samples from oral squamous cell carcinoma (OSCC) patients and controls was carried out using immunome protein microarrays. Autoantibodies (AAbs) against 1,628 proteins (including controls) were assayed (**Supplementary Table 3**). Principal Component Analysis (PCA) revealed the clustering of control samples compared to the OSCC samples (**Figure 1A** **).** We identified a total of 101 autoantibodies with differential levels between OSCC and control groups, including 59 upregulated (log_2_ FC>=0.58 or 1.5-fold) and 42 downregulated (log_2_ FC<=-0.58 or 0.66-fold) (**Figures 1B, C**). Of these, autoantibodies against 25 proteins were significantly increased (**Supplementary Table 4**). In comparison, autoantibodies against 28 proteins were significantly decreased (**Supplementary Table 5**) (p-value threshold of < 0.05, penetrance fold change difference of ≥ 2 or ≤ -2) and Frequency Differential ≥ 1 or ≤ -1).

### 3.1 Autoantibodies identified in oral cancer

**Figure 2:**
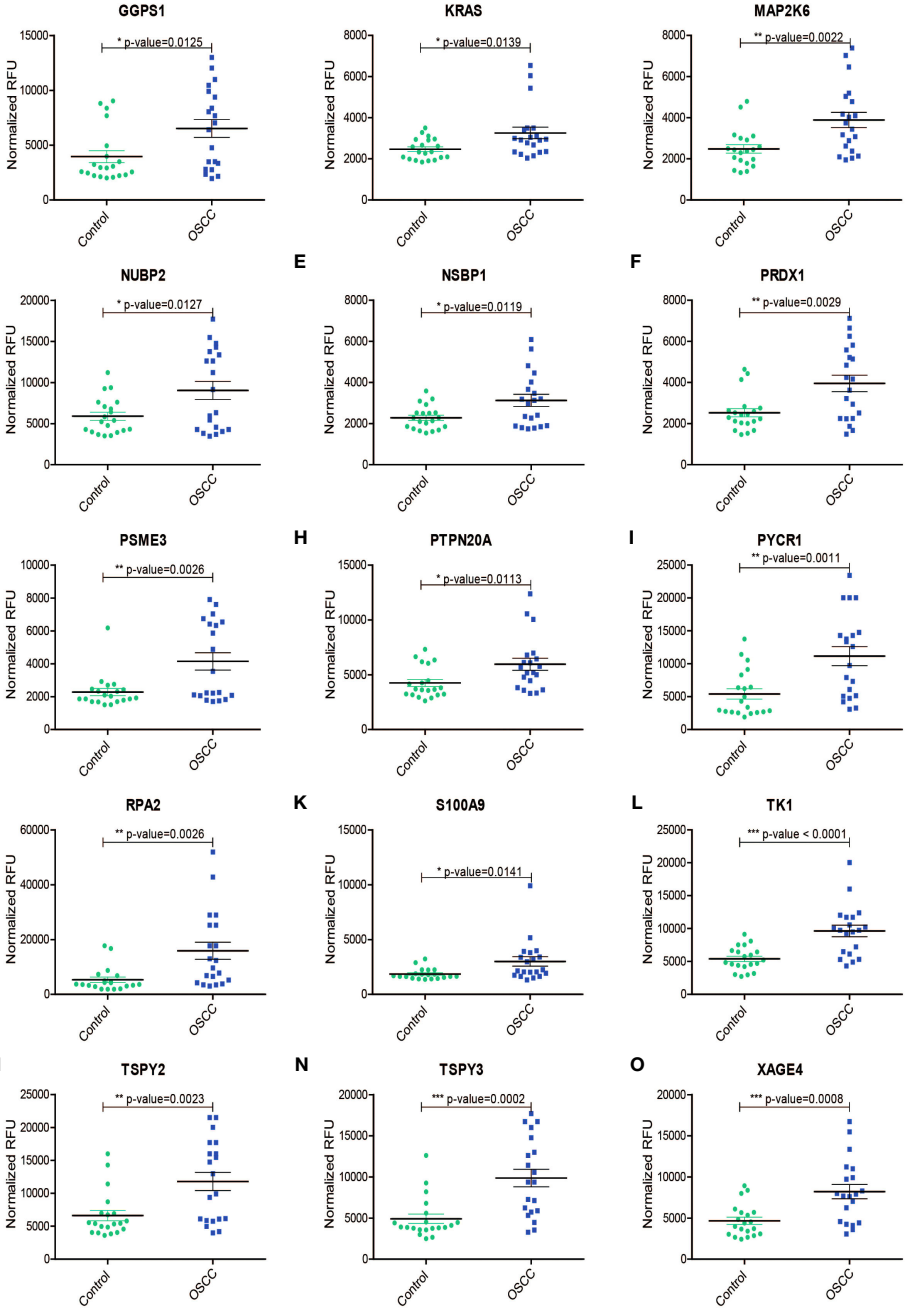
Increased levels (Normalized RFU) of protein autoantibodies in serum samples from patients with oral squamous cell carcinoma (OSCC)as compared to those from healthy volunteers (Control) for **(A)** GGPS1, **(B)** KRAS, **(C)** MAP2K6, **(D)** NUBP2, **(E)** NSBP1, **(F)** PRDX1, **(G)** PSME3, **(H)** PTPN20A, **(I)** PYCR1, **(J)** RPA2, **(K)** S100A9, **(L)** TK1, **(M)** TSPY2, **(N)** TSPY3, **(O)** XAGE4. *, P ≤ 0.05; **, P ≤ 0.01; ***, P ≤ 0.001.

Of the 25 AAbs observed to be increased in the serum of OSCC samples (**Figures 2A–O**), a subset of candidate antigens have been described as overexpressed in the context of oral cancer, including GGPS1 (32), KRAS (33), MAP2K6 (34), PRDX1 (35), PSME3 (36), S100A9 (37), and TAGLN (38). Further increased expression of tumor-associated antigens has been reported in other cancers for a subset of antigens such as RPA2 (39–41), PYCR1 (42, 43), and TK1 (44–46). Interestingly, no published evidence was available concerning candidate antigens- NUBP2, PTPN20, TSPY2, TSPY3, and XAGE4 against which we observed significantly increased levels of AAbs. The findings are summarized in **Table 1**. The potentially novel candidate markers have distinct functions and activities. Further, we also identified AAbs against 28 candidate antigens to be decreased in the serum of OSCC samples compared to controls (**Supplementary Figures 1A–O**). We correlated the increased/decreased levels of autoantibodies with the site of oral cancer (**Supplementary Figures 2**, **3**). We also queried the expression of upregulated genes in normal, primary and metastatic cases from the TCGA HNSCC transcriptome data, across clinical stages and mapped survival curves for the expression (**Supplementary Figures 4**-**9**). We chose to compare with the TCGA HNSCC cohort of 528 samples as the majority of the cases are resected from the oral cavity (72.4%) (29).

**Table 1:** Details of autoantibodies increased in OSCC serum as compared to controls.

Candidates that have not been found to be reported in the context of oral cancer					
AAb^#^	Protein Name	Fold-change*	p Value	Remarks	
NUBP2	Nucleotide binding protein 2	2.81	0.015	No relevant data in the literature
PTPN20	Protein tyrosine phosphatase non-receptor type 20	2.70	0.012	No relevant data in the literature
TSPY2	Testis specific protein, Y-linked 2	3.17	0.003	No relevant data in the literature
TSPY3	Testis specific protein, Y-linked 3	3.42	3E-04	No relevant data in the literature
XAGE4	XAGE-4 protein	3.25	0.001	No relevant data in the literature
Candidate markers that have been previously reported in oral cancer (positive controls)					
**AAb^#^**	**Protein Name**	Fold-change*	**p Value**	**Remarks**
GGPS1	Geranylgeranyl diphosphate synthase 1	3.03	0.013	GGPS expression levels correlated with tumor sensitivity to HMGCR inhibitor, pitavastatin (32).
KRAS	KRAS proto-oncogene, GTPase	2.54	0.016	KRAS expression was found to be an important determinant for HNSCC cell proliferation. Amplification of non-mutated KRAS was found to contribute to tumor growth (33).
MAP2K6	Mitogen-activated protein kinase kinase 6	2.80	0.002	MAPK26 was a potential candidate for mediating cisplatin resistance in OSCC (34)
PRDX1	Peroxiredoxin 1	2.72	0.004	Increased overexpression of the RAB2A and PRDX1 gene observed in OSCC (35).
PSME3	Proteasome (prosome, macropain) activator subunit 3	3.14	0.003	Overexpression is associated with adverse prognosis in patients with OSCC. The aberrant expression of PA28γ may contribute to the pathogenesis and progression of OSCC (36).
S100A9	S100 calcium binding protein A9	4.06	0.017	Expression of S100A9 and S100A8 in brush biopsies was able to differentiate between normal mucosa from premalignant and oral squamous cell carcinoma cells (37).
TAGLN	Transgelin	2.86	0.5	Increased tissue/salivary transgelin transcripts predicted poor prognosis in OSCC patients undergoing surgery (38).
Candidates that have been previously reported in the context of cancers					
**AAb^#^**	**Protein Name**	Fold-change*	**p Value**	**Remarks**
RPA2	Replication protein A2, 32kDa	6.35	0.004	Overexpression and phosphorylation of RPA2 were reported in several cancers, including esophageal (39), bladder (40), and ovarian cancers (41).
PYCR1	Pyrroline-5-carboxylate reductase 1	3.86	0.001	PYCR1 is induced by a shortage of proline precursors, and its suppression attenuated kidney cancer cell proliferation when proline was limiting. High PYCR1 is frequently observed in invasive breast carcinoma (42), and increased expression in prostate cancer (43).
TK1	Thymidine kinase 1, soluble	3.33	2E-04	Serum TK1 expression up in breast, prostate (44), lung (45), esophageal (46) cancers.

**#AAb**, Autoantibody.

*Fold-change, Penetrance Fold Change Difference.

Based on these data, several of these candidates could serve as potential biomarkers for OSCC.

### 3.2 The Fe/S cluster assembly protein NUBP2 is expressed in a large majority of OSCC patients

**Figure 3:**
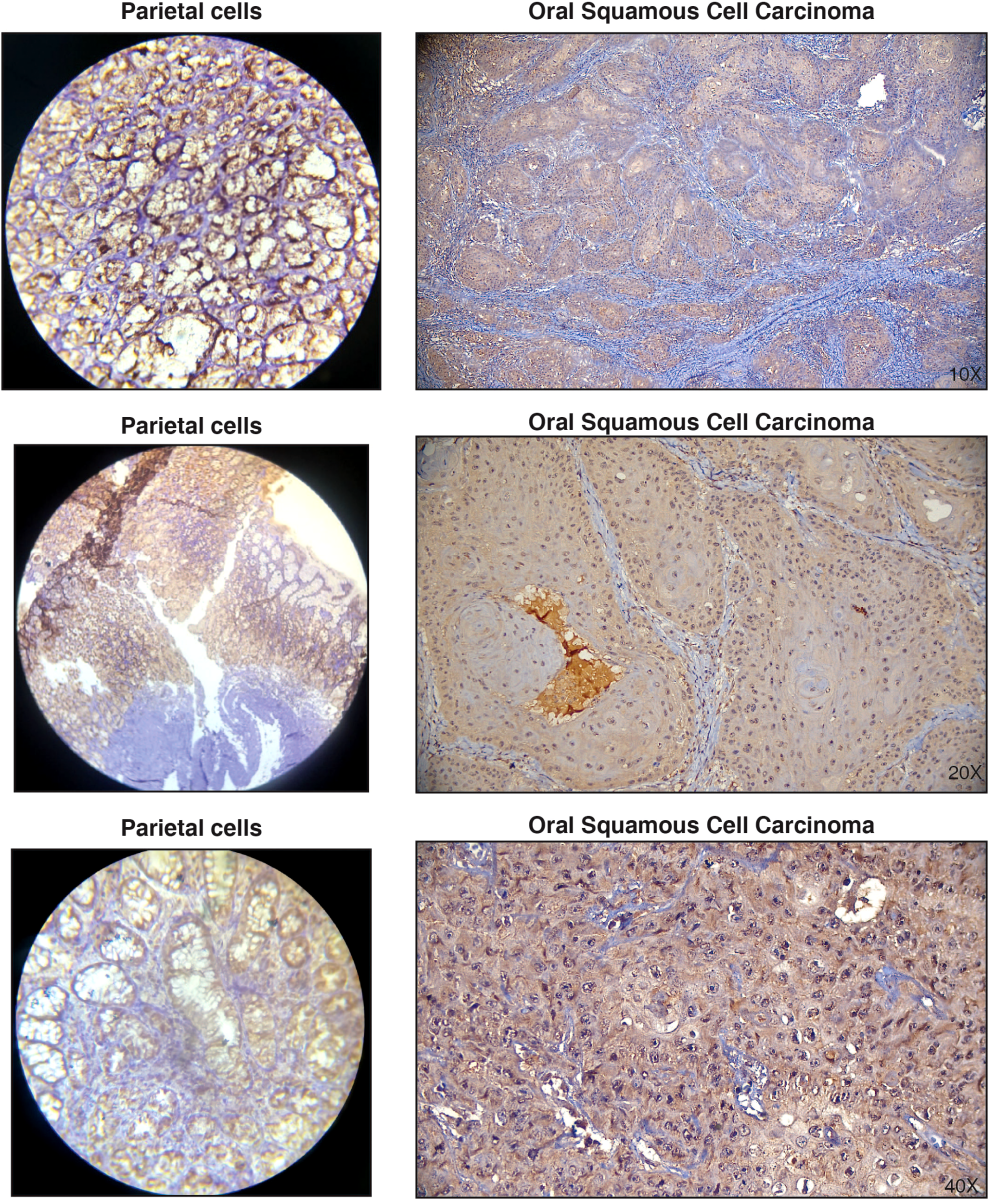
Representative immunohistochemistry images showing positive staining of NUBP2 in Oral squamous cell carcinoma. Parietal cells were used as positive controls.

It has been well established that protein overexpression in the tumor tissue causes autoantibody production (47, 48). Therefore, we chose to assess if the increase in serum autoantibodies we identified was due to increased protein expression in the tumor tissue. To achieve this, we chose to validate the candidate antigen- NUBP2, a Fe/S cluster assembly protein. To the best of our knowledge, NUBP2 has not been reported in the context of OSCC. The expression level of NUBP2 was assessed using immunohistochemical analysis in a panel of 25 OSCC cases on tissue microarrays (**Figure 3**). Although a large majority of OSCC cases (72%) showed positive staining, NUBP2 positivity did not significantly correlate with patient age, gender, tumor site, tumor size, or lymph node metastasis (**Table 2**).

**Table 2:** Immunohistochemical results for NUBP2.

	Immunohistochemistry		
	Positive N(%)	Negative N(%)	Significance (Fisher’s exact test)
**NUBP2 staining (n=25)**	18 (72%)	7 (28%)	
Age			
>45 (n=17)	11 (64.7%)	6 (35.3%)	**.362, NS**
<45 (n=8)	7 (87.5%)	1 (12.5%)	
Gender			
Male (n=17)	12 (70.6%)	5 (29.4%)	**.607, NS**
Female (n=8)	6 (75%)	2 (25%)	
Site of tumor			
Buccal mucosa (n=12)	8 (66.7%)	4 (33.3%)	**.130, NS**
Floor of mouth (n=6)	3 (50%)	3 (50%)	
Tongue (n=7)	7 (100%)	0 (0%)	
Lymph node metastasis (N)			
Positive (n=13)	11 (84.6%)	2 (15.4%)	**.202, NS**
Negative (n=12)	7 (58.3%)	5 (41.7%)	
Tumor Size (T)			
T1 (n=6)	5 (84.6%)	1 (16.7%)	**.923, NS**
T2 (n=10)	7 (70.0%)	3 (30.0%)	
T3 (n=7)	5 (71.4%)	2 (28.6%)	
T4 (n=2)	1 (50.0%)	1 (50.0%)	

*NS, Not significant.

### 3.3 NUBP2 is differentially expressed in HNSCC, and its expression correlates with survival

**Figure 4:**
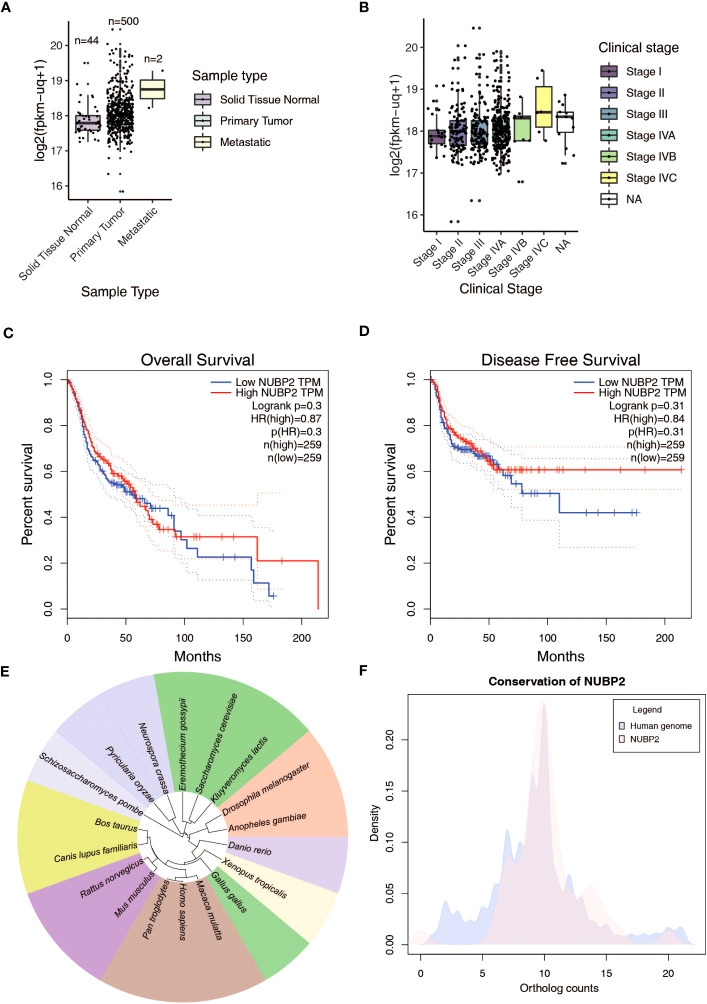
**(A)** *NUBP2* expression across sample types in GDC TCGA Head and Neck Cancer RNASeq-HTSeq data from Xena Functional Genomics Explorer. **(B)** *NUBP2* expression across clinical stages in GDC TCGA Head and Neck Cancer RNASeq-HTSeq data from Xena Functional Genomics Explorer. Kaplan Meier plots showing correlation of NUBP2 expression with **(C)** Overall Survival and **(D)** Disease Free survival in patients with HNSCC from TCGA. **(E)** Figure showing sequence similarity of NUBP2 across various species. **(F)** Conservation of NUBP2 across species.

We were unable to assess from the immunohistochemistry experiments if NUBP2 positivity translated to NUBP2 overexpression due to the unavailability of normal tissue samples due to ethical reasons. Therefore, we chose to look at publicly available datasets, including TCGA, which had previously carried out a comprehensive investigation of head and neck squamous cell carcinomas (HNSCC) (49). We queried the expression of NUBP2 in normal, primary and metastatic HNSCC cases from the TCGA transcriptome data (**Figure 4A**). *NUBP2* expression was found to be higher in primary and metastatic HNSCC cases as compared to normal, with the highest expression observed in the metastatic HNSCC cases. However, this finding was insignificant as the number of metastatic HNSCC samples in the TCGA was too small. Further, the *NUBP2* expression correlated with HNSCC clinical stages (**Figure 4B**) with slightly elevated levels observed in stages IVB and IVC. These findings, however, need to be confirmed in a larger cohort of patients. *NUBP2* expression correlated with the overall patient and disease-free survival in the TCGA cohort (**Figures 4C, D**). Higher NUBP2 expression correlated with poor overall survival, and better disease-free survival.

A literature search on the functional role of NUBP2 indicated that it was relatively less studied. Sequence conservation analysis revealed the protein was well conserved across species (**Figures 4E, F**). Since NUBP2 is one of the major cytosolic components of the Fe/S cluster assembly and is relatively less characterized, we sought to analyze the expression of other components of the cytosolic and nuclear Fe/S cluster assembly in oral cancer (50) and TCGA head and neck cancer (49) data (**Supplementary Figure 10A**). Several members of the cytosolic/nuclear Fe/S cluster assembly, including CIAO1, CIAO2B, CIAPIN1, MMS19, NUBP1, and NUBP2, were differentially expressed in these datasets (**Supplementary Figure 10B**). These findings suggest that the proteins belonging to the Fe/S cluster assembly are differentially expressed in OSCC and warrant further investigation into the mechanism and functional implication of this finding.

## 4 Discussion

Tumor autoantibodies (TAAbs) have been known to be produced in response to tumor antigens. Several mechanisms for TAAb production in cancer have been proposed (48), including overexpression of tumor antigens which could include any protein present at increased levels in tumors as compared to normal physiological levels (47). Consequently, protein overexpression in tumors could lead to the formation of TAAbs. TAAbs have a variety of applications. They have been described as potential diagnostic biomarkers that are stable and detectable before the onset of clinical symptoms (51, 52). Further, they can serve as prognostic

markers, as in the case of early-stage non-small cell lung cancer (NSCLC) (21), or as predictive markers for immunotherapy response, for example, to observe anti-PD1 therapy response in alveolar soft part sarcoma (NASPS), NSCLC and lymphoma (53). Autoantibody profiling is being increasingly used to study TAAb signatures in various cancers, including lung (21), melanoma (54), gastric (55), thyroid (56), and ovarian cancers (57).

The current study used the autoantibody profiling approach to identify potential biomarkers for OSCC. Among the TAAbs identified, we found NUBP2, a cytosolic Fe/S cluster assembly protein (58), to be promising, as no previous references with respect to OSCC were found. Further, we found high NUBP2 positivity in OSCC cases and aberrant expression of NUBP2 and other cytosolic Fe/S cluster assembly members in previous datasets. Iron-sulfur (Fe/S) clusters are small inorganic protein cofactors that are involved in fundamental biochemical processes such as the electron transport chain, maintenance of genomic stability, RNA modification, gene regulation, and DNA repair, amongst others (59, 60). Several proteins participate in the assembly of these Fe/S clusters in the mitochondria, cytoplasm, and nucleus. The Fe/S cluster assembly pathway has been found to play important roles in tumor cell biology and has been suggested as a potential therapeutic target for cancer (61). Downregulation of the Fe/S cluster protein assembly -ISCU (iron-sulfur cluster assembly enzyme) by miR-210 induces Reactive Oxygen Species (ROS) production in hypoxia, a preferential shift to glycolysis, increased lactate production, and enhanced cell survival in tumor cells (62). Further, tumors with reduced ISCU had a worse prognosis in breast cancer and HNSCC. Fe/S cluster assembly protein MMS19 was suggested to play a role in DNA repair, regulation of genome stability factors, and telomere maintenance, suggesting its importance in cancer biology (63, 64). MMS 19 expression was associated with metastasis and therapy response in esophageal squamous cell carcinoma (ESCC) (65). MMS19 was also identified as a predictive marker of adjuvant therapy response in NSCLC (66). Members of the cytosolic Fe/S assembly pathway- MMS19 and CIAO2B were found to be essential for replication stress tolerance of cancer cells towards Chk1 and ATR inhibition (67). These studies highlight the importance of the Fe/S cluster assembly proteins in cancer biology and warrant more investigation into NUBP2 and other members of the family in the context of cancers.

Besides, NUBP2, AAbs of TK1, PSME3, and RPA2 were increased in OSCC as compared to control samples and require further validation in a larger cohort of patients. Curiously, AAbs for proteins involved in cell cycle regulation (CCNB1, CDK1, CDK16, CDK8) and cytokines/chemokines (IL8RB, CCR5, CXCR4, CXCR6) were found to be decreased in the serum of OSCC patients as compared to control samples. The significance of decreased AAbs in cancer is uncertain and requires further investigation.

## 5 Conclusions

Much of the global burden of OSCC is localized to Asian countries, and most cases are diagnosed at advanced stages. Therefore, it is essential to screen vulnerable populations to aid in early diagnosis and subsequent early treatment of OSCC. The discovery of differential levels of AAbs in OSCC in the current study paves the way for their potential use as biomarkers for screening, diagnosis, or prognosis of the disease using liquid biopsies. Besides being stable and persistent, circulating AAbs can indicate a tumor’s immune state. The validity of the AAbs identified in the current study will need validation in a larger cohort of patients. Further investigations will be required to determine the functional role of these AAbs in cancer.

## Data availability statement

All data generated or analyzed during this study are included in this published article and its supplementary information files.

## Ethics statement

The studies involving human participants were reviewed and approved by Yenepoya (Deemed to be University) Ethics Committee Mangalore (#2013/149 dated 24/07/2013 and #2016/239 dated 12/11/2016). The patients/participants provided their written informed consent to participate in this study.

## Author contributions

Conceptualization: RA, PR, YS. Methodology: RA, JP, SP, YS. Formal analysis and investigation: RA, JP, YS. Writing - original draft preparation: YS. Writing - review and editing: RA, SP, YS. Funding acquisition: RA, PR. Supervision: RA and YS. All authors contributed to the article and approved the submitted version.
