# Supplementary material for: Serum autoantibody profiling of oral squamous cell carcinoma patients reveals NUBP2 as a potential diagnostic marker
Source: Front Oncol. 2023 Sep 22;13:1167691. doi: 10.3389/fonc.2023.1167691 (PMC10556692; doi:10.3389/fonc.2023.1167691)

Supplementary Material

Serum autoantibody profiling of oral squamous cell carcinoma patients reveals NUBP2 as a potential diagnostic marker

Riaz Abdulla1,*, Jofy Devasia Puthenpurackal1, Sneha M. Pinto2,3, P.D. Rekha2, Yashwanth Subbannayya2,3*

1 Department of Oral Pathology and Microbiology, Yenepoya Dental College, Yenepoya (Deemed to be University), Mangalore 575018, India

2 Yenepoya Research Centre, Yenepoya (Deemed to be University), Mangalore, 575018, India

3 School of Biosciences and Medicine, Faculty of Biosciences and Medicine, University of Surrey, Guildford, UK

*** Correspondence:** Corresponding Author: y.subbannayya@surrey.ac.uk (Yashwanth Subbannayya), rizdent@yenepoya.edu.in (Riaz Abdulla)

Supplementary Tables

Supplementary Table 1. Details of patient samples used for immunome autoantibody profiling

Supplementary Table 2. Details of patient samples used for immunohistochemical validation and correlation with IHC results

Supplementary Table 3. Normalized RFU (Relative Fluorescence) data for protein autoantibodies identified in serum samples of patients with oral squamous cell carcinoma (case) and volunteers (control)

Supplementary Table 4. Normalized RFU (Relative Fluorescence) for significant protein autoantibodies found to be increased in serum samples of patients with oral squamous cell carcinoma (case) and volunteers (control) and p-value < 0.05

Supplementary Table 5. Normalized RFU (Relative Fluorescence) for significant protein autoantibodies found to be decreased in serum samples of patients with oral squamous cell carcinoma (case) and volunteers (control) and p-value < 0.05

Supplementary Figures

Supplementary figure 1: Decreased levels (Normalized RFU) of protein autoantibodies in serum samples from patients with oral squamous cell carcinoma (OSCC)as compared to those from healthy volunteers (Control) for **A.** ABI1 , **B.** BRSK1 , **C.** BTG1 , **D.** CCNB1 , **E.** CCR5, **F.** CDC2, **G.** CDK16 (PCTK1), **H.** CDK8 , **I.** CKMT2, **J.** COPB2, **K.** CXCR4, **L.** CXCR6, **M.** CXCR2/IL8RB, **N.** PRC1, **O.** RPS6KA3

Supplementary figure 2: Correlation of increased levels (Normalized RFU) of protein autoantibodies across site of oral cancer for in serum samples from patients with oral squamous cell carcinoma (OSCC)as compared to those from healthy volunteers (Control) for **A.** GGPS1 , **B.** KRAS, **C.** MAP2K6, **D.** NUBP2, **E.** NSBP1 , **F.** PRDX1 ,**G.** PSME3 ,**H.** PTPN20A, **I.** PYCR1, **J.** RPA2, **K.** S100A9, **L.** TK1, **M.** TSPY2, **N.** TSPY3, **O.** XAGE4

Supplementary figure 3: Correlation of decreased levels (Normalized RFU) of protein autoantibodies across site of oral cancer for in serum samples from patients with oral squamous cell carcinoma (OSCC)as compared to those from healthy volunteers (Control) for **A.** ABI1 , **B.** BRSK1 , **C.** BTG1 , **D.** CCNB1 , **E.**CCR5 , **F.** CDC2, **G.** CDK16 (PCTK1), **H.** CDK8 , **I.** CKMT2, **J.** COPB2, **K.** CXCR4, **L.** CXCR6, **M.** CXCR2/IL8RB, **N.** PRC1, **O.** RPS6KA3

Supplementary figure 4: Expression of upregulated candidates **A.** GGPS1 , **B.** KRAS, **C.** MAP2K6, **D.** HMGN5 (NSBP1), **E.** NUBP2, **F.** PRDX1 ,**G.** PSME3 ,**H.** PTPN20A, and **I.** PYCR1 across sample types in GDC TCGA Head and Neck Cancer RNASeq-HTSeq data from Xena Functional Genomics Explorer

Supplementary figure 5: Expression of upregulated candidates **A.** RPA2, **B.** S100A9, **C.** TK1, **D.** TSPY2, and **E** TSPY3, across sample types in GDC TCGA Head and Neck Cancer RNASeq-HTSeq data from Xena Functional Genomics Explorer

Supplementary figure 6: : Expression of upregulated candidates **A.** GGPS1 , **B.** KRAS, **C.** MAP2K6, **D.** HMGN5 (NSBP1), **E.** NUBP2, **F.** PRDX1 ,**G.** PSME3 ,**H.** PTPN20A, and **I.** PYCR1 across clinical stages in GDC TCGA Head and Neck Cancer RNASeq-HTSeq data from Xena Functional Genomics Explorer.

Supplementary figure 7: : Expression of upregulated candidates **A.** RPA2, **B.** S100A9, **C.** TK1, **D.** TSPY2, and **E** TSPY3, across clinical stages in GDC TCGA Head and Neck Cancer RNASeq-HTSeq data from Xena Functional Genomics Explorer.

Supplementary figure 8: : Kaplan Meier plots showing correlation of expression of upregulated candidates **A.** GGPS1 , **B.** KRAS, **C.** MAP2K6, **D.** HMGN5 (NSBP1), **E.** PRDX1 ,**F.** PSME3 ,**G.** PTPN20A, **H.** PYCR1, **I.** RPA2, **J.** S100A9, and **K.** TK1, with Disease Free survival in patients with Head and neck cancer from TCGA

Supplementary figure 9: : Kaplan Meier plots showing correlation of expression of upregulated candidates **A.** GGPS1 , **B.** KRAS, **C.** MAP2K6, **D.** HMGN5 (NSBP1), **E.** PRDX1 ,**F.** PSME3 ,**G.** PTPN20A, **H.** PYCR1, **I.** RPA2, **J.** S100A9, and **K.** TK1, with Overall survival in patients with Head and neck cancer from TCGA

Supplementary figure 10: A. Members of the nuclear/cytosolic components of the Fe/S cluster assembly **B.** Expression of the nuclear/cytosolic components of the Fe/S cluster assembly in OSCC (Mumtaz *et al.* (2022)) and HNSCC (TCGA (2015)) datasets.

Supplementary figure 1

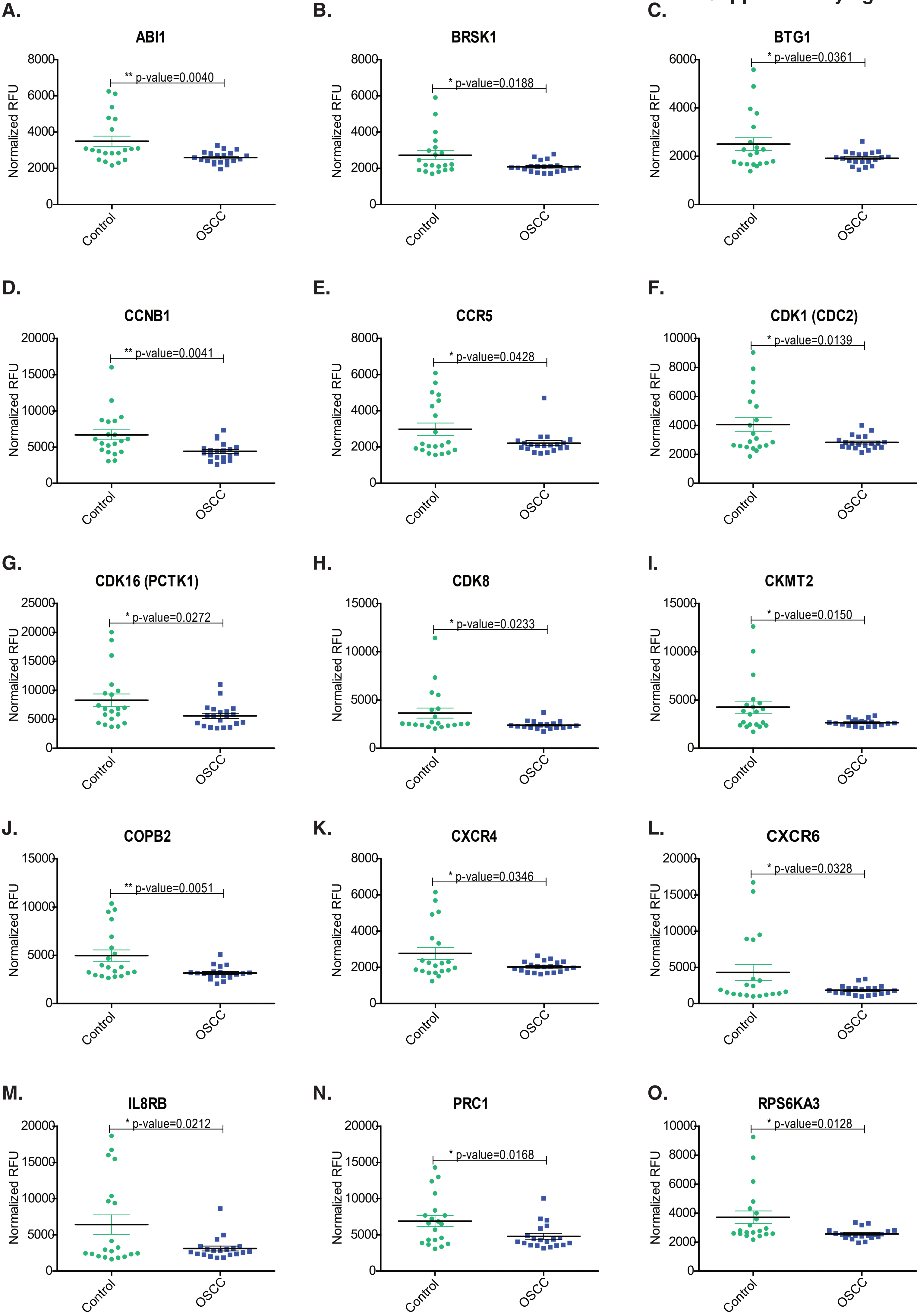

Supplementary figure 2

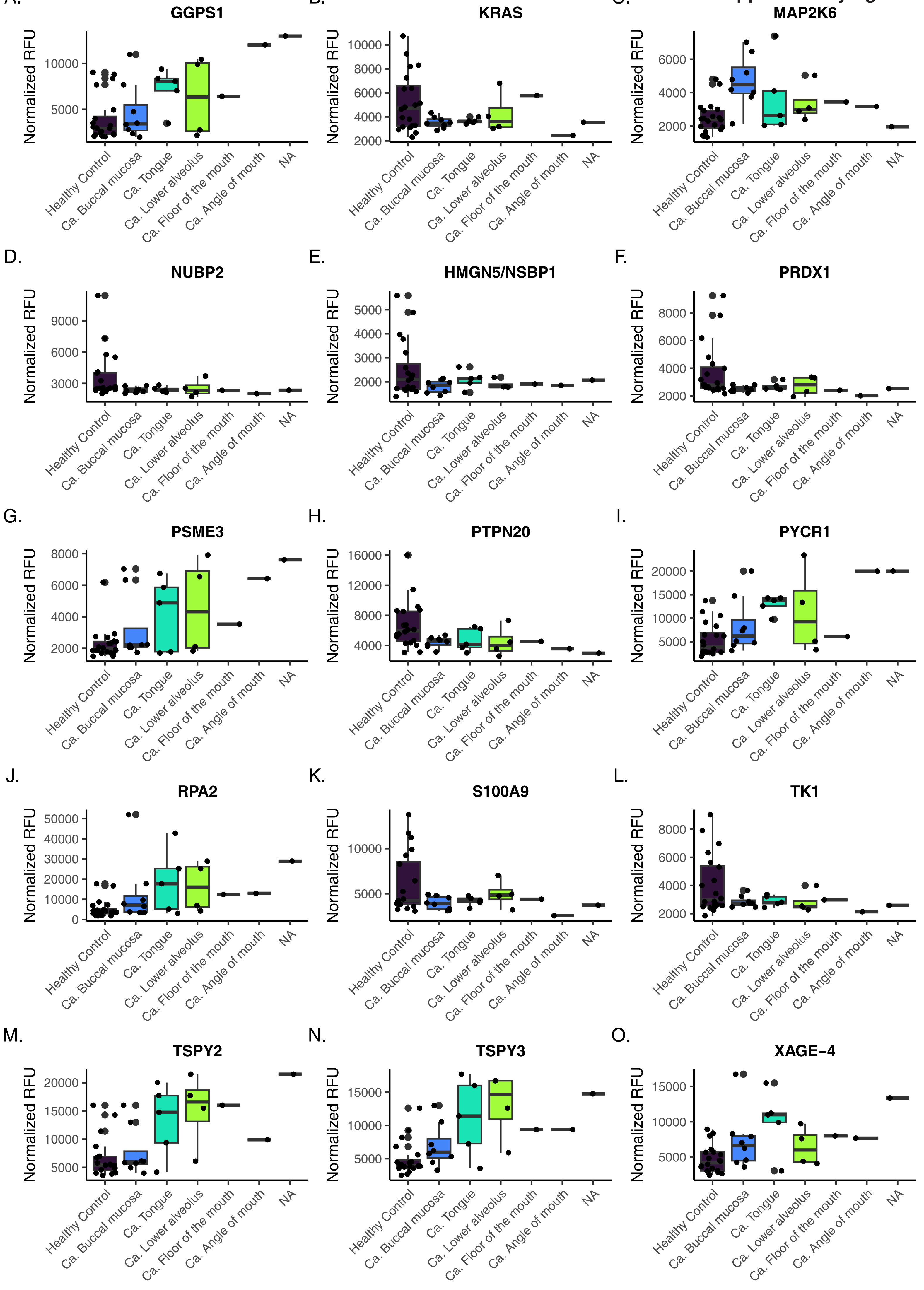

Supplementary figure 3

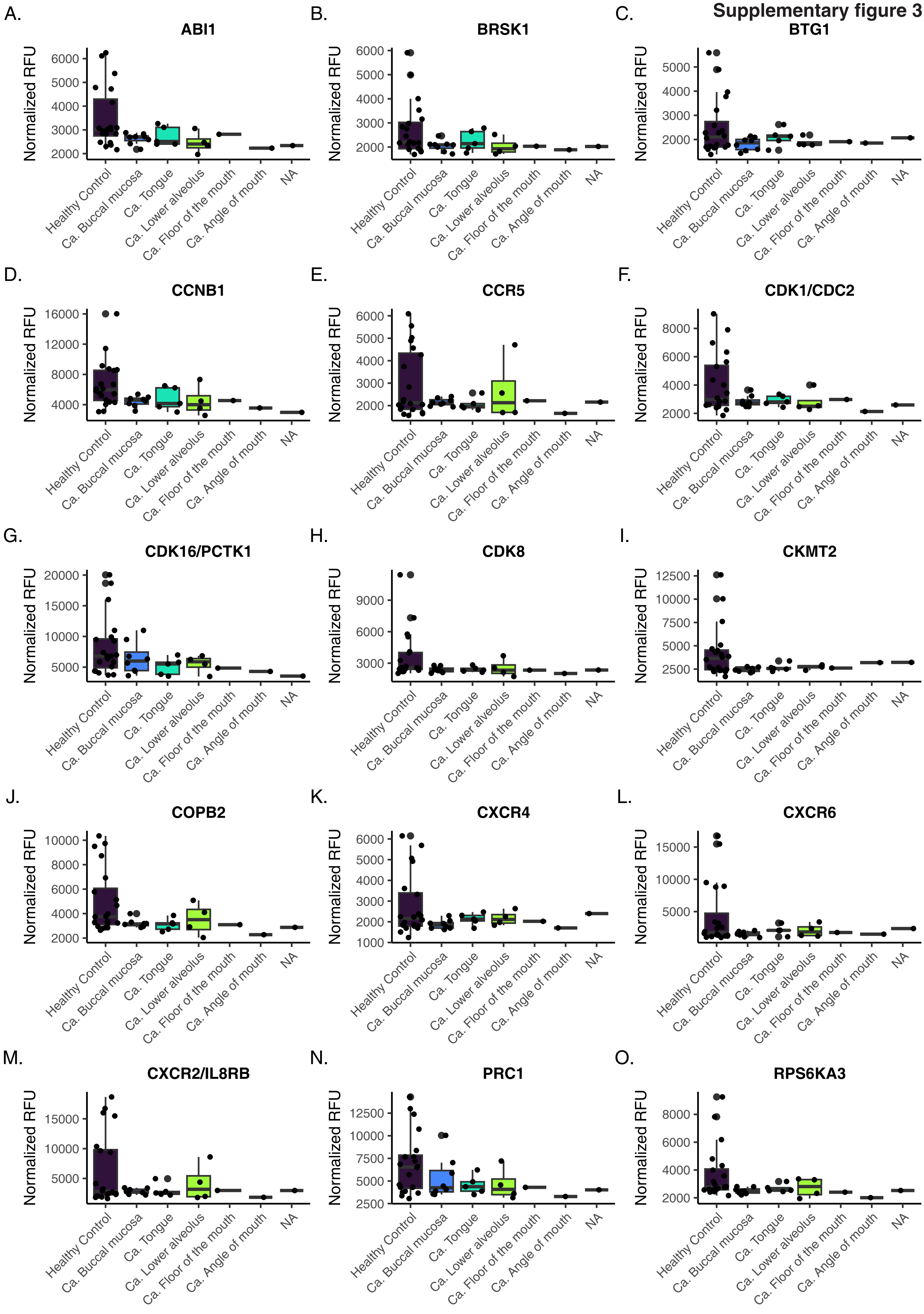

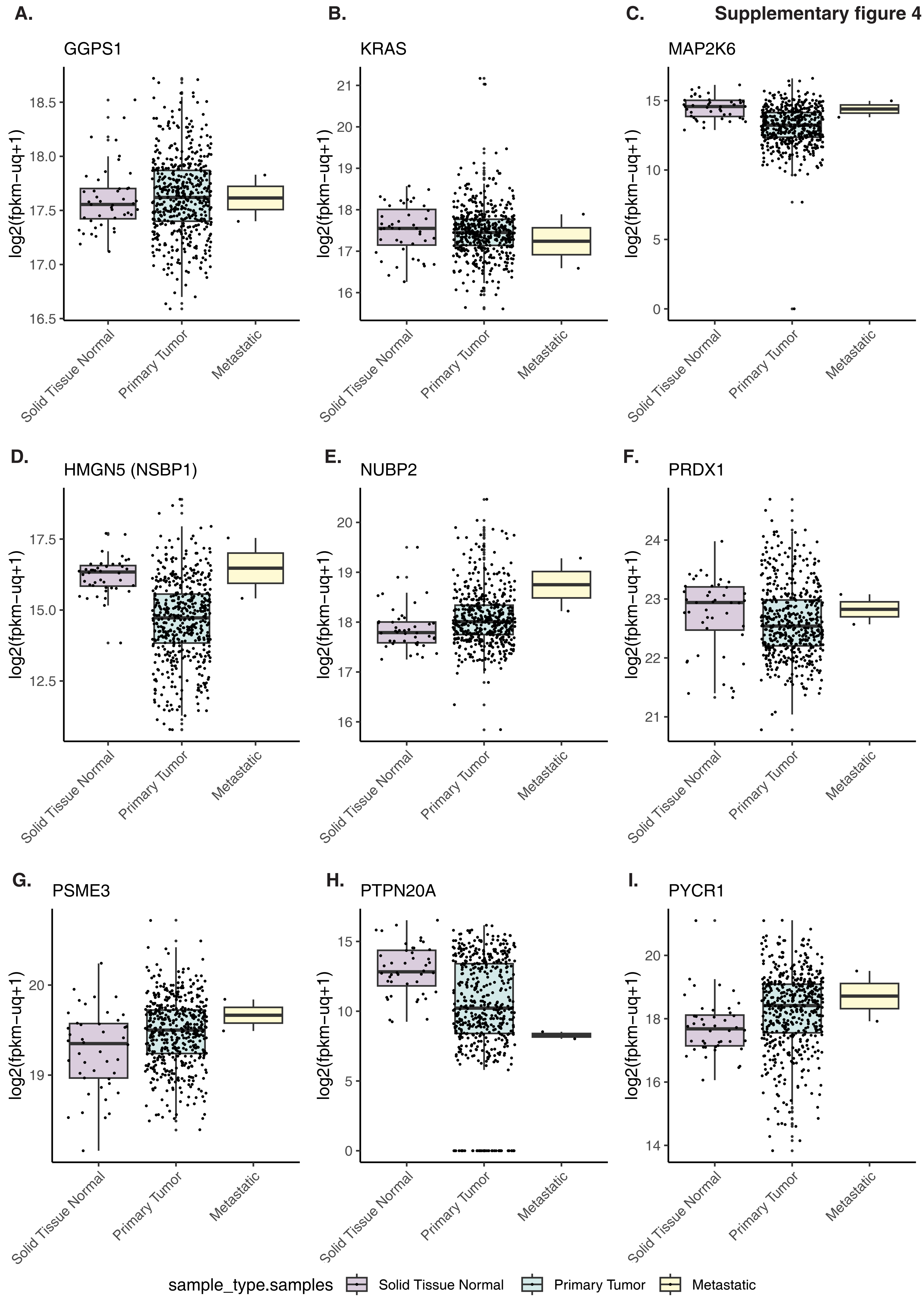
Supplementary figure 4
A.
GGPS1
B.
KRAS
C.
MAP2K6
D.
HMGN5 (NSBP1)
E.
NUBP2
F.
PRDX1
G.
PSME3
H.
PTPN20A
I.
PYCR1
log2(fpkm−uq+1)
Solid Tissue Normal
Primary Tumor
Metastatic
sample_type.samples

Supplementary figure 5

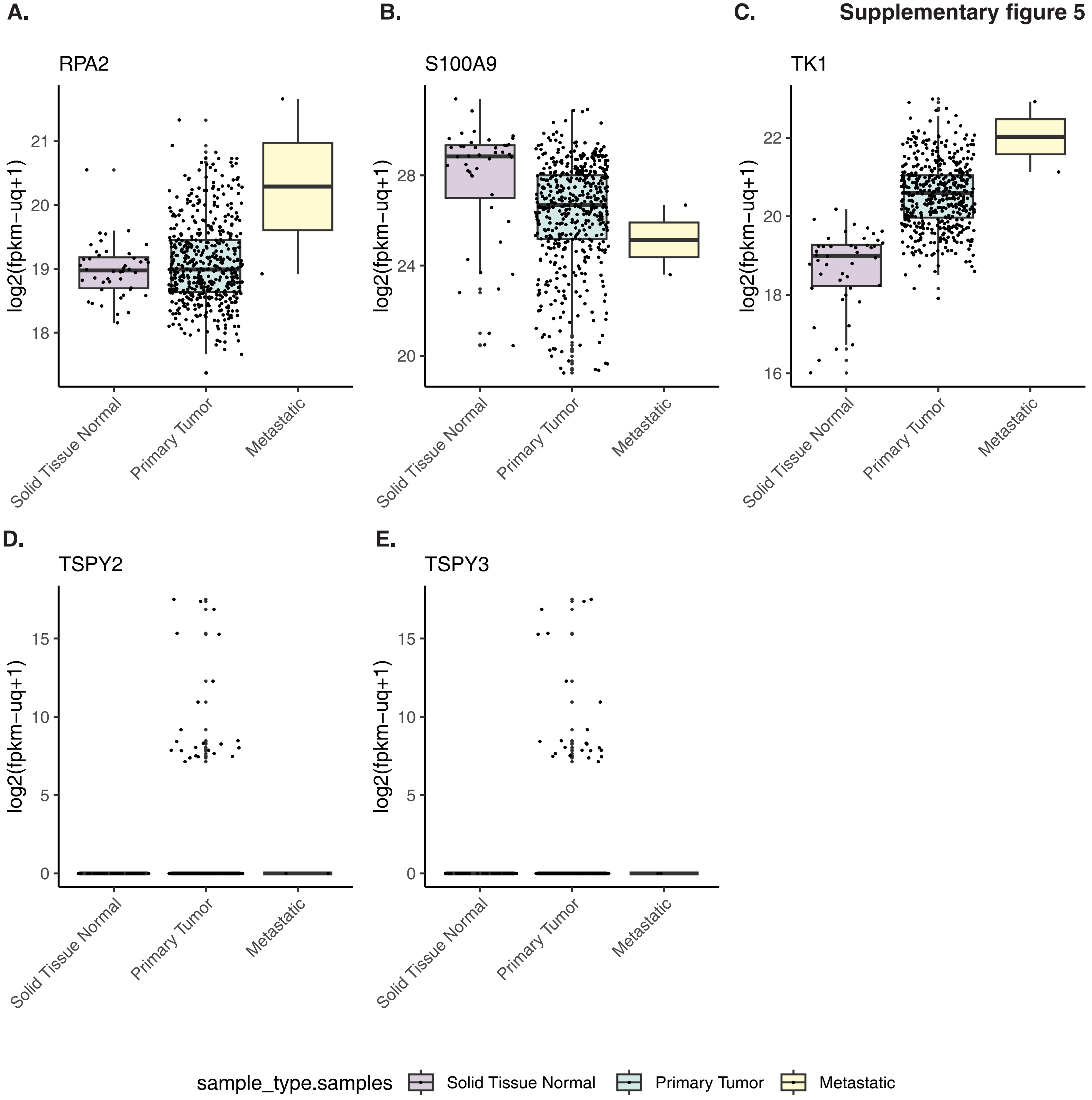

Supplementary figure 6

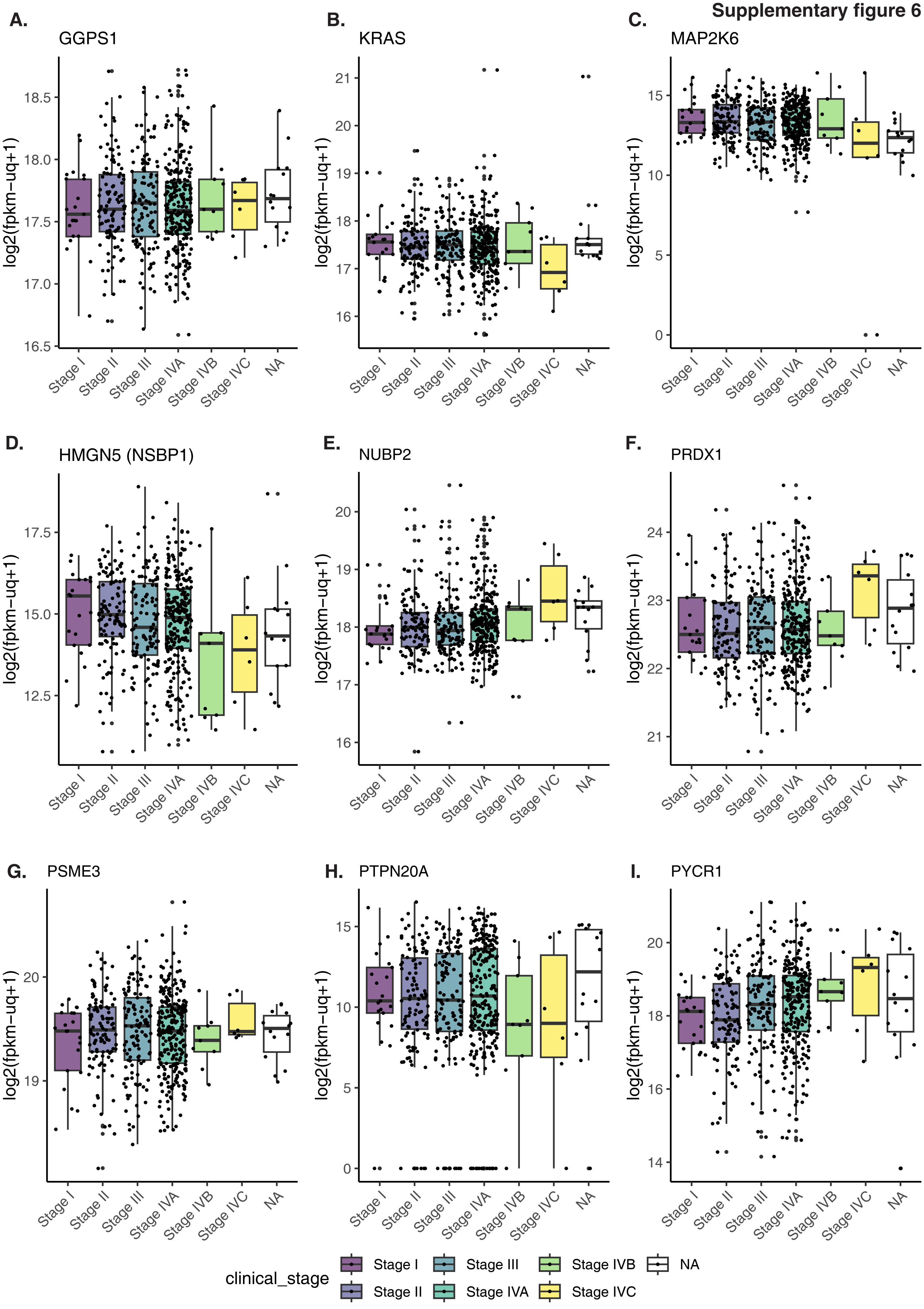

Supplementary figure 7

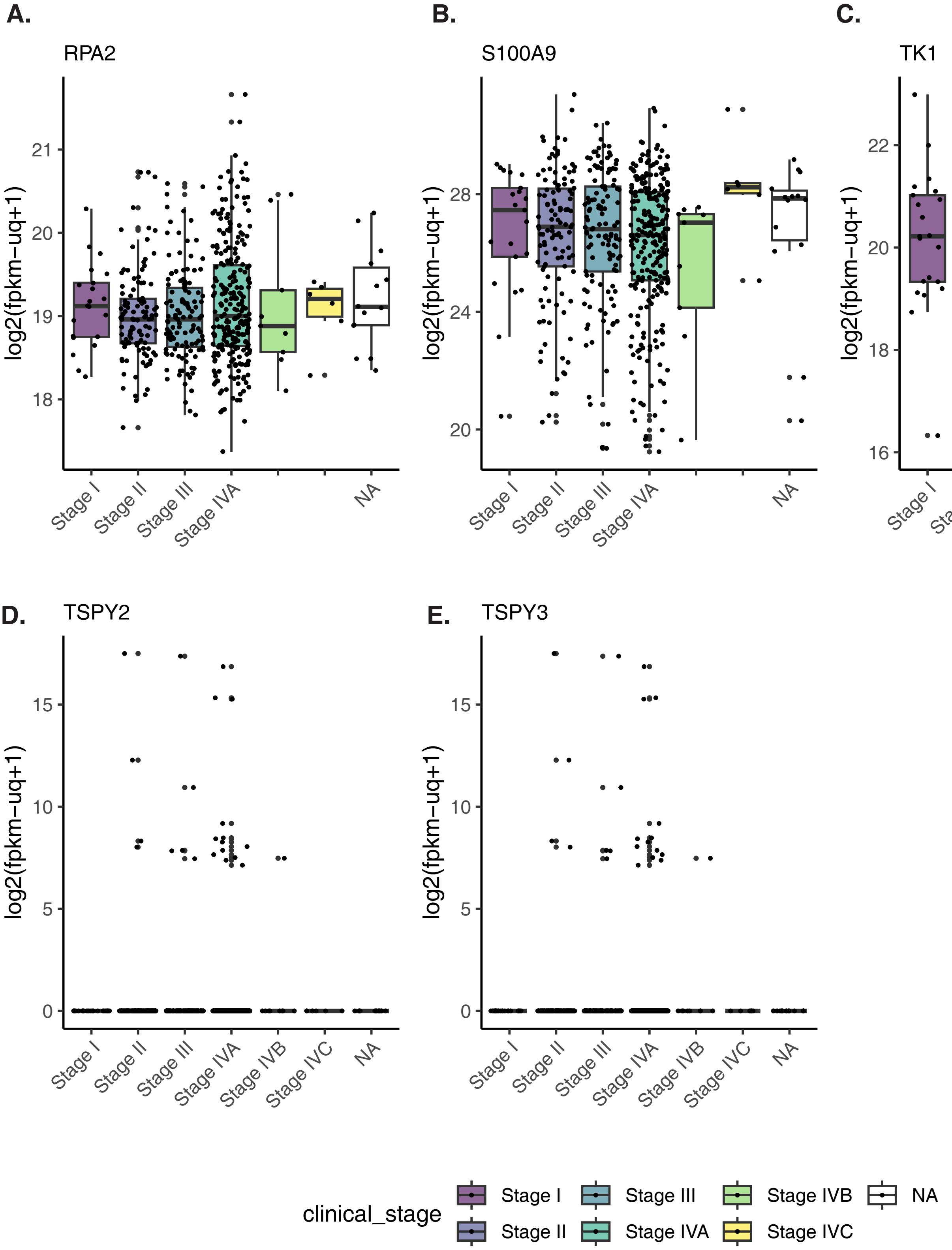

Supplementary figure 8

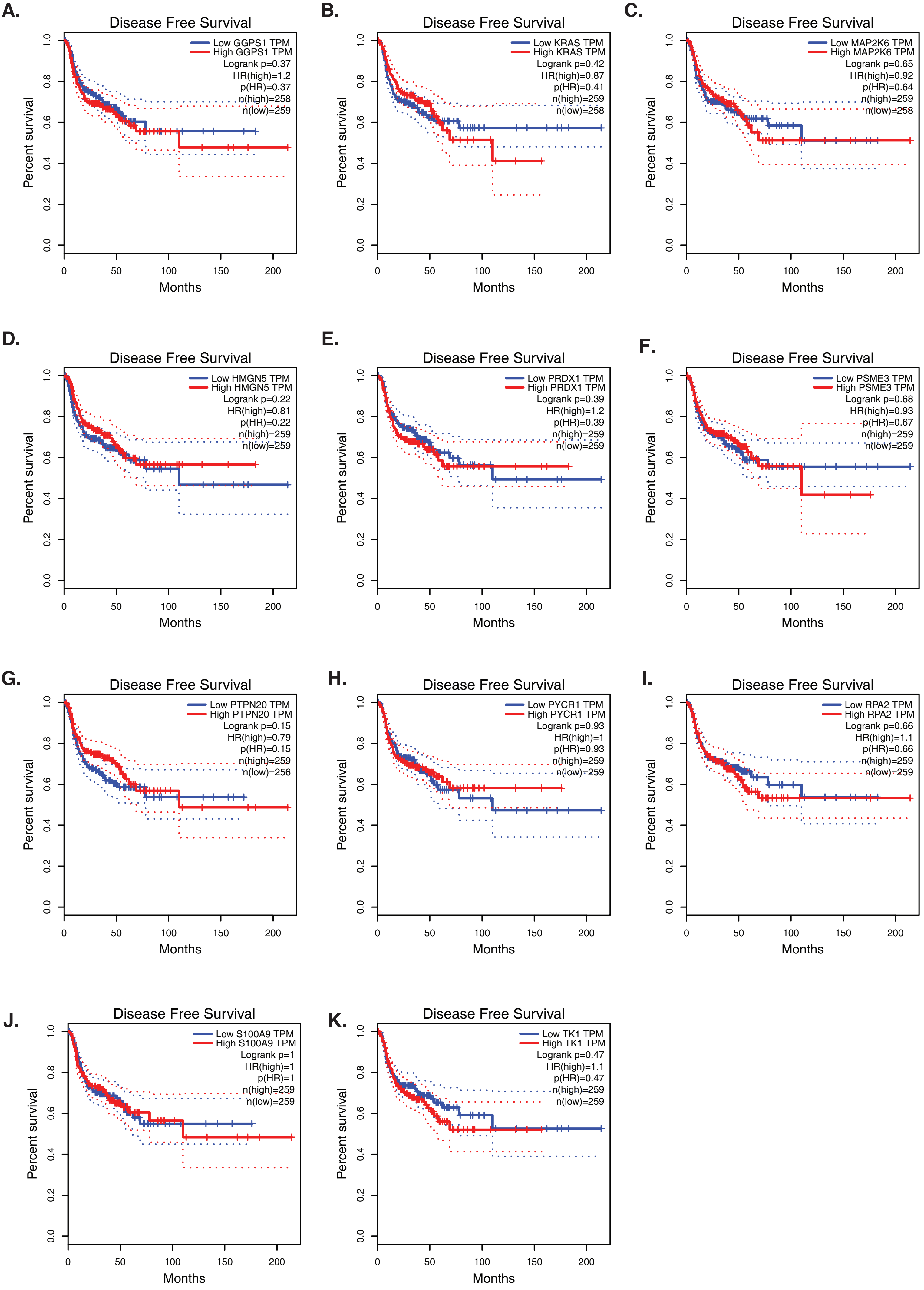

Supplementary figure 9

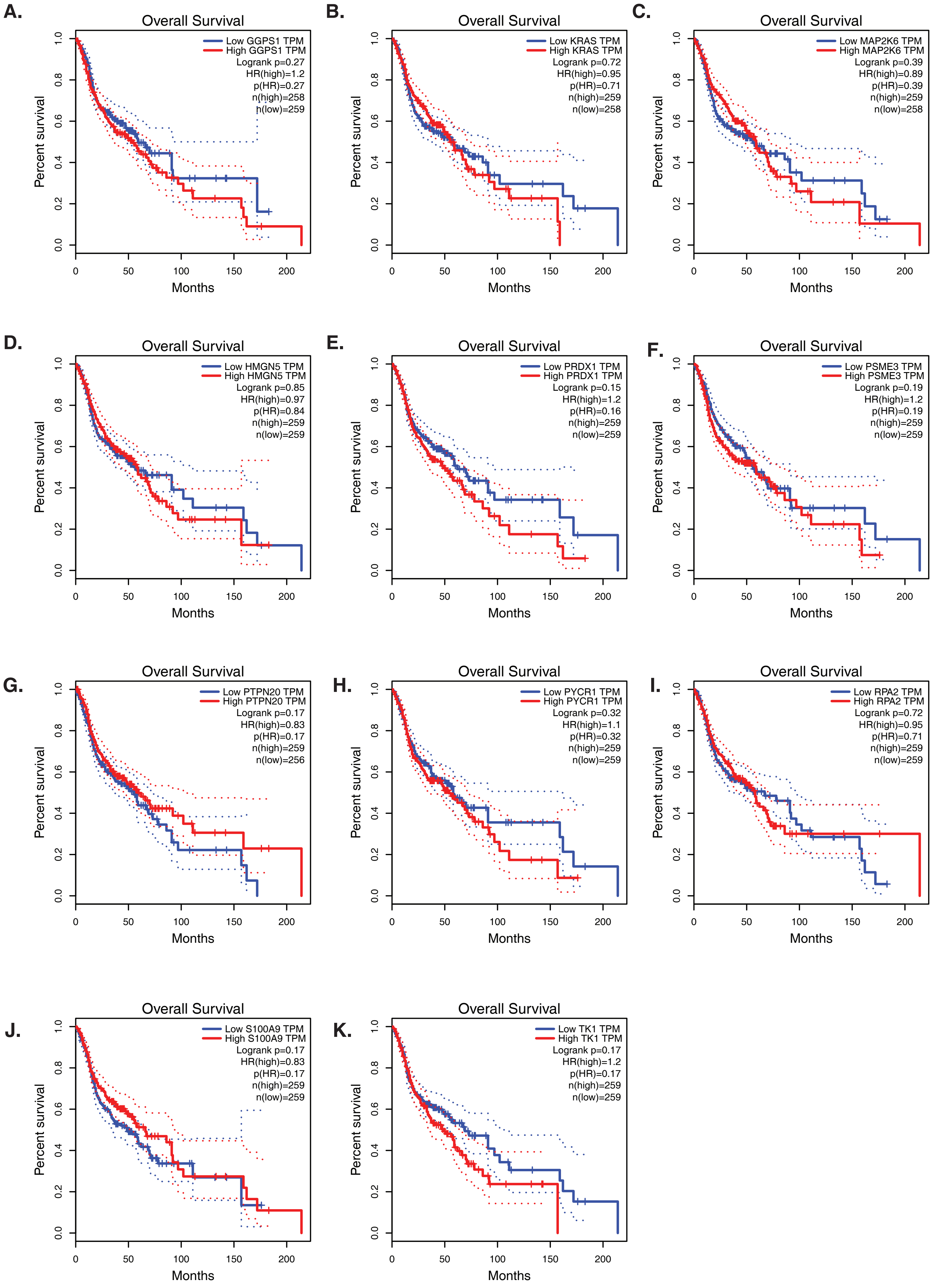

Supplementary figure 10

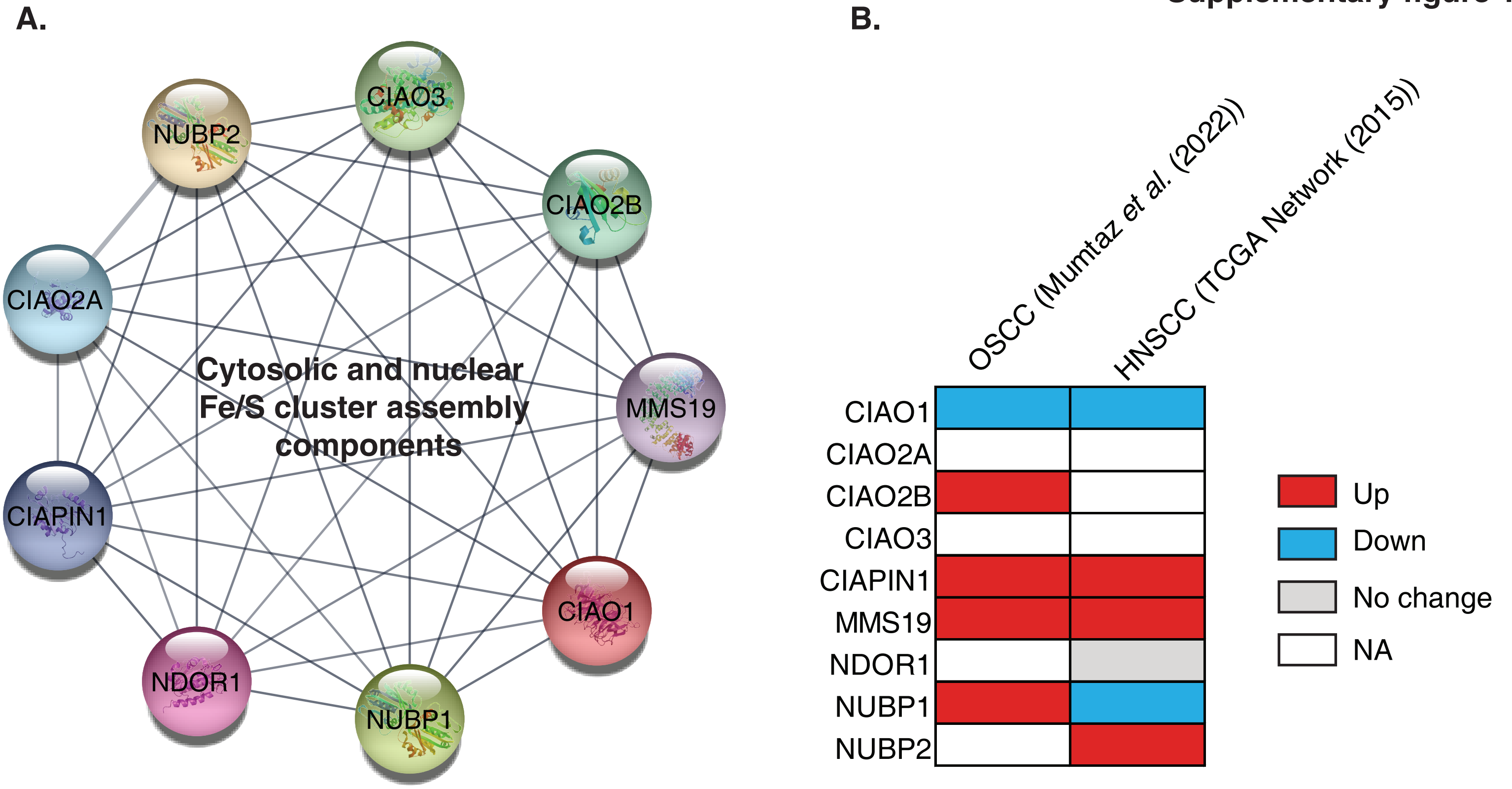

Supplement: Supplementary file 1 [file DataSheet_1.pdf]
